# The data universe of structural biology

**DOI:** 10.1107/S205225252000562X

**Published:** 2020-05-28

**Authors:** Helen M. Berman, Brinda Vallat, Catherine L. Lawson

**Affiliations:** a Department of Chemistry and Chemical Biology, Rutgers, The State University of New Jersey, Piscataway, NJ 08854, USA; b Department of Biological Sciences and Bridge Institute, University of Southern California, Los Angeles, CA 90089, USA; c Institute for Quantitative Biomedicine, Rutgers, The State University of New Jersey, Piscataway, NJ 08854, USA

**Keywords:** Protein Data Bank, structural biology, X-ray crystallography, data resources, data standards

## Abstract

The Protein Data Bank (PDB) has grown from a small data resource for crystallographers to a worldwide resource serving structural biology. The history of the growth of the PDB and the role that the community has played in developing standards and policies are described. This article also illustrates how other biophysics communities are collaborating with the worldwide PDB to create a network of interoperating data resources. This network will expand the capabilities of structural biology and enable the determination and archiving of increasingly complex structures.

## Abstract 2

The history of the growth of the Protein Data Bank and the role that the community has played in developing standards and policies are described.

## 1. Introduction   

Crystallographers have a long tradition of effective data management practices. It is intriguing to speculate on the origins of these practices. Perhaps the requirement for ordered crystals carries over into a need for ordered results. Or perhaps it is a consequence of the fact that crystallographic experiments generate large volumes of data, yielding definitive results that are utilized by many other scientists. From its inception, the International Union of Crystallography (IUCr) took a leadership role in promoting data standards; one of the stated objectives of the IUCr is ‘to facilitate standardization of methods, units, nomenclatures and symbols’ (https://www.iucr.org). This high level of standardization has enabled us to efficiently turn the relatively high volume of data produced by crystallographic experiments first into information, and then into knowledge. Another objective of the IUCr ‘to promote international cooperation in crystallography’, beyond creating the necessary standards, created a framework for data sharing.

Data sharing in the crystallographic community has been achieved by the development of databases, some of which are summarized in a recent article by Bruno *et al.* (2017 ▸). One of the first data resources to be established was the Powder Diffraction File by the International Centre for Diffraction Data (Faber & Fawcett, 2002 ▸; Kabekkodu *et al.*, 2002 ▸). Established in 1941, it currently houses more than one million datasets. The Cambridge Structural Database (CSD), established in 1965 by Olga Kennard, currently contains over one million small molecule structures (Groom *et al.*, 2016 ▸). Inspired in part by these resources, the Protein Data Bank (PDB) was established in 1971 to serve as an archive for the structures of biomacromolecules (Protein Data Bank, 1971 ▸). Since that time, the PDB has evolved from a data archive for biological macromolecular crystal structures to a resource for all structural biology methods. In this review, we describe this evolution with an emphasis on how the community has worked together to develop standards and policies for data sharing.

## 2. The Protein Data Bank   

### 2.1. Early history   

The Protein Data Bank began as a grassroots movement in the 1960s when the very first protein structures were published (Kendrew *et al.*, 1960 ▸; Perutz *et al.*, 1960 ▸). In an era when punched cards and magnetic tapes were the media for data storage and the post office was the only way to distribute information, the task of sending data to a colleague was overwhelming. At the same time, there was an increasing interest in protein folding and it was recognized that protein structure data could be enormously useful in tackling the challenge of structure prediction (Levinthal, 1968 ▸). Starting in the 1960s, a series of informal meetings were held among the producers and potential users of atomic coordinate data. At the *Cold Spring Harbor Symposium* on protein structure held in June 1971 (Cold Spring Harbor Laboratory, 1972 ▸), Walter Hamilton offered to set up the Protein Data Bank at Brookhaven National Laboratory. He immediately flew to England and made an agreement with Olga Kennard, the head and founder of the CSD, to collaborate on such an enterprise. The announcement of the PDB appeared in *Nature New Biology* in October 1971 (Protein Data Bank, 1971 ▸). Hamilton worked with Edgar Meyer and Helen Berman to set up the PDB; after Hamilton’s untimely death in 1973, Tom Koetzle became the head of the PDB.

**Figure 1:**
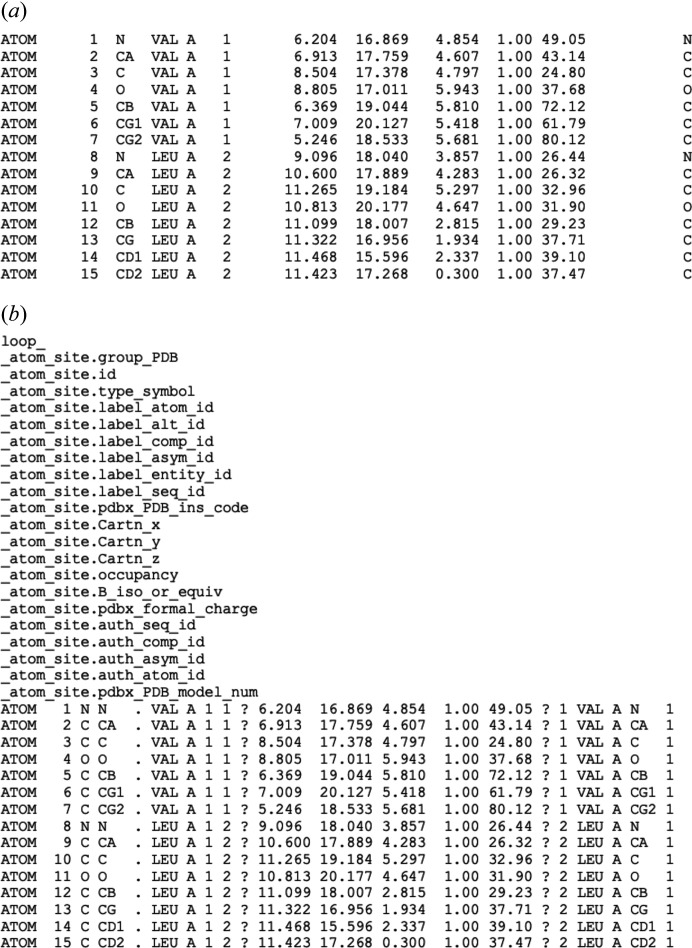
Formats for representation of atomic coordinates. (*a*) PDB format. All data items are in fixed-sized fields and definitions are implicit. (*b*) mmCIF format. The names of the data items as defined in the mmCIF dictionary are listed first using a loop directive. The values of the data items then follow in a tabular form. This representation enables mmCIF to be flexible, self-consistent and software compatible.

In the early days, data submission was entirely voluntary. To encourage data deposition, Tom Koetzle wrote letters to protein crystallographers making them aware of the resource. The PDB Format (Bernstein *et al.*, 1977 ▸) (Fig. 1 ▸), based on the 80-column punched card, contained data fields for the coordinates and metadata describing the crystallographic experiment and the chemistry of the molecules in the crystal. Data distribution was accomplished using magnetic tapes and a newsletter announced the PDB holdings (Protein Data Bank, 1974 ▸).

The earliest structures in the PDB were determined using X-ray crystallography. In 1985, the first structure determined using nuclear magnetic resonance (NMR) spectroscopy was published (Williamson *et al.*, 1985 ▸), and in 1990 the first structure determined using three-dimensional electron microscopy (3DEM) was incorporated into the PDB (Henderson *et al.*, 1990 ▸).

The PDB was managed by the Brookhaven National Laboratory from 1971 until 1999. In 1999, the Research Collaboratory for Structural Bioinformatics (RCSB) (Berman *et al.*, 2000 ▸) – a consortium consisting of researchers from San Diego Supercomputer Center (SDSC), Rutgers and the National Institute of Standards and Technology (NIST) – began to manage the archive. In 2003, the Worldwide PDB (wwPDB) was created to formally recognize the global reach of the PDB (Berman *et al.*, 2003 ▸). The initial partners were RCSB PDB, the Macromolecular Structure Database [MSD (Boutselakis *et al.*, 2003 ▸); now PDBe] and PDB Japan [PDBj; (Nakamura *et al.*, 2002 ▸)]. The wwPDB partners formalized an agreement that there would be a single global archive with data that are freely and publicly available. Informed by advice from a Scientific Advisory Committee, the wwPDB sets the standards and procedures for processing and distributing data.

### 2.2. Deposition guidelines   

The 1980s saw a new kind of activism in the crystallographic community. Many felt very strongly that data sharing should be a condition of publication. Among them was Richard Dickerson (Barinaga, 1989 ▸) who wrote letters to colleagues and to journals promoting the idea that coordinate data should be deposited into the PDB. Fred Richards circulated a petition signed by almost 200 colleagues urging the same. In that same period, several different committees were set up to study the issue. One organized by the IUCr Commission on Biological Macromolecules discussed in detail what data should be deposited; after several years of discussion and deliberation, guidelines were published (International Union of Crystallography, 1989 ▸). It was recommended that coordinates should be submitted to the PDB; deposition of structure factors was optional. Hold periods were allowed before data release. Once these guidelines were in place and backed by strong sentiments in the community, the campaign to require data deposition succeeded. Although it took some time, virtually all journals that publish macromolecular structures now require data deposition into the PDB.

### 2.3. mmCIF standard   

During the 1980s a new format called the Crystallographic Information File (CIF) was developed (Hall *et al.*, 1991 ▸). It is a self-defining text format that contains the key definitions for most aspects of crystallographic experiment. Its design is suitable for small molecules and allows for easy validation of these structures. The CIF format was adopted by the IUCr and American Chemical Society journals. In the early 1990s, the IUCr set up a new committee to create a CIF-like format for macromolecular structures. It soon became apparent that because of the complexity of macromolecular structures, the syntax of CIFs would not be suitable. A new variant called the Macromolecular Crystallographic Information File (mmCIF) was created (Bourne *et al.*, 1997 ▸).

mmCIF is a self-defining format that specifies the standards for representing macromolecular structures (Fitzgerald *et al.*, 2005 ▸). These standards include definitions for describing the experimental procedures, the chemistry of the components and the results of a biomacromolecular crystallographic structure determination. mmCIF also provides mechanisms to enforce data consistency, which is important for archiving. A comparison of coordinate records in the PDB and mmCIF is shown in Fig. 1 ▸.

mmCIF has been designed to be extensible. Over time, the wwPDB has extended mmCIF to build the PDBx/mmCIF metadata framework [http://mmcif.wwpdb.org; Westbrook *et al.* (2005 ▸); Westbrook & Fitzgerald (2009 ▸)] which enables archiving of structural models obtained from X-ray diffraction, NMR and 3DEM experiments. In addition to definitions for representing macromolecular structures, the framework also includes descriptions of the supporting metadata such as information about source organisms, samples, workflows, authors, citations, software and model quality metrics.

Because of its syntax, mmCIF allows for the creation of relational databases and it was clear that it would be useful for storing PDB data. However, the pushback on mmCIF by the community was very strong. The PDB format was simple and human readable. It was used by hundreds of software programs for structure determination and analysis. However, the 80-column format meant strict limitations on the number of atoms that could be stored within a single file and large structures had to be split into multiple files. mmCIF was first adopted by the Nucleic Acid Database (Berman *et al.*, 1992 ▸) and by the PDB when its management was taken over by the RCSB in 1999, but it was not until 2011 that crystallographic software developers agreed to adopt mmCIF. From this point PDBx/mmCIF became the master format for the PDB. All of the very large structures that had needed to be split into multiple files in the PDB format were then converted to single mmCIF files; access to these complex structures is now greatly simplified. A PDBx/mmCIF working group was set up under the auspices of the wwPDB to enable the use of the format in major software packages. Starting in 2019, all X-ray structure depositions are required to be in mmCIF format (Adams *et al.*, 2019 ▸).

### 2.4. Validation   

In the very early days of the PDB, the primary focus of annotation was to ensure that data were formatted correctly and that there were no obvious errors. In time, validation procedures were set up to check the geometry, nomenclature and chemistry of the coordinate files. Among the items checked is the stereochemistry, which includes valence geometry, dihedral angles, planarity and chirality. Non bonded contacts as well as crystallographic and non-crystallographic symmetry are assessed. The primary sequence of the polymer is checked against sequence databases and the geometry of the small molecules is evaluated.

Notably absent in these early assessments were checks against the primary data. In 2000, the IUCr recommended that structure factors be a requirement of deposition and enforced this requirement for its journals (IUCr Commission on Biological Macromolecules, 2000 ▸). Although this was endorsed by many in the community (Wlodawer, 2007 ▸), it was not until 2008 that the deposition of structure factors became mandatory. This requirement, plus suspicions that there were some fraudulent structures in the PDB (Berman *et al.*, 2010 ▸), led the wwPDB to convene an X-ray Validation Task Force (VTF). The X-ray VTF, led by Randy Read and consisting of thought leaders in crystallographic methods, studied possible checks that could be done on the full complement of data. The entire corpus of data (70,000) was run against these checks to assess outliers. A final set of recommendations were made (Read *et al.*, 2011 ▸).

The X-ray VTF recommended that a small set of validation data be presented in an easily understood format, with comparisons made with both the full PDB archive and the structure resolution class. The suggested validation criteria included measures that evaluate the fit of the structure to the experimental data [*R*_free_ and real-space residual *Z* scores (Brünger, 1992 ▸; Kleywegt *et al.*, 2004 ▸)], assess the quality of the coordinates [clashes, protein backbone, side-chain rotamers and buried unsatisfied hydrogen-bonds (Laskowski *et al.*, 1993 ▸; Chen *et al.*, 2010 ▸; Dunbrack & Cohen, 1997 ▸; Hooft *et al.*, 1996 ▸)] and check the crystal lattice for underpacking (Sheffler & Baker, 2009 ▸). The VTF developed a novel ‘sliders’ representation that compactly displays a structure’s score values for each of the key criteria, as well as its percentile rank in the archive, and compares it with other structures in the same resolution range. They also listed criteria that should be flagged for review in any incoming PDB structure entry: poor overall geometry or extreme local geometry distortion, inverted chirality, structure factor intensity outliers, incorrect data labels, missed symmetry, missed twinning, incomplete structure, poor ligand density or geometry, and inconsistent carbohydrate nomenclature.

In addition to the X-ray VTF, an NMR VTF (Montelione *et al.*, 2013 ▸) and 3DEM VTF (Henderson *et al.*, 2012 ▸) have been established and have produced their respective recommendations for validation.

**Figure 2:**
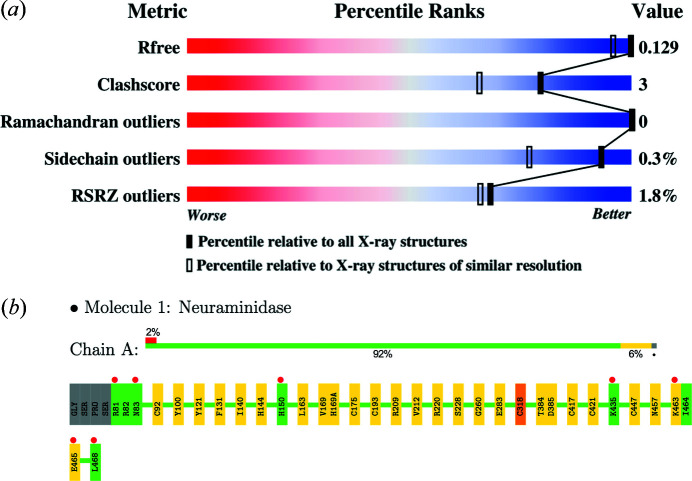
Key elements of the wwPDB validation report for X-ray structures are shown for PDB entry 6pzd, a recent crystal structure of Influenza A neuraminidase, determined at 1.12 Å (Zhu *et al.*, 2019 ▸). (*a*) Graphical display of key metrics (‘sliders’). For each metric, two percentile ranks are calculated: an absolute rank with respect to the entire PDB archive and a relative rank with respect to structures determined at similar resolution. Slider markers in the blue region on the right are indicative of a high-quality structure. Lower-quality structures have the markers in the red region on the left. (*b*) Residue property plot: residues are color-coded green if no issues are detected, yellow if there are outliers for one criterion (*e.g.* unusual bond lengths), orange if there are outliers for two criteria (*e.g.* unusual bond lengths and clashes) and red for three or more criteria. A horizontal stack bar plot presents the fraction of residues with each color code. Unmodeled regions of the chain, if present, are represented by a gray segment. The upper red bar indicates the fraction of residues with poor fit to the electron density.

Concurrent with the work of the VTFs, the wwPDB partners began a project to create a unified system for deposition, curation and validation of structures that have been submitted to the PDB. The recommendations of the VTFs became the basis of the validation suite (Gore *et al.*, 2017 ▸) for the new system, called *OneDep* (Young *et al.*, 2017 ▸). The validation report includes most of the indicators recommended by the X-ray VTF including a slider [Fig. 2 ▸(*a*)], various geometric checks and graphical summaries of chain quality [Fig. 2 ▸(*b*)]. Many journals now require authors to submit PDB validation reports with their manuscripts. Thus, the structural biology community has set a very high bar for responsible reporting of research results.

### 2.5. Current state of the PDB   

**Figure 3:**
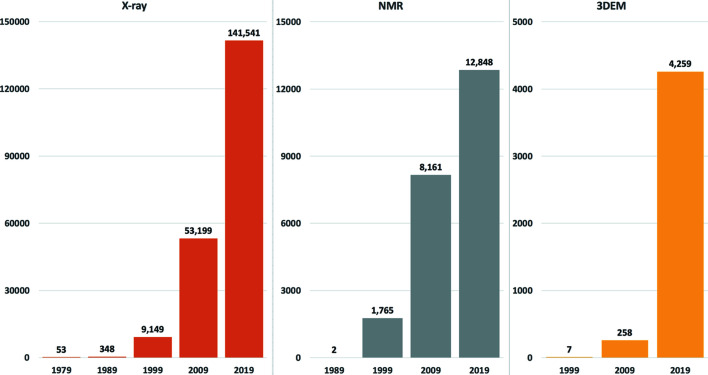
Cumulative holdings of the PDB at the end of each decade for each of the three major structure determination methods, X-ray crystallography, NMR and 3DEM, respectively. 3DEM methods include structures determined by electron microscopy (single-particle, helical, subtomogram averaging and tomography) and electron crystallography.

The rate of growth of PDB holdings has increased dramatically (wwPDB Consortium, 2019 ▸). From seven relatively small crystal structures there are now more than 160,000. Fig. 3 ▸ shows the growth charts for structures determined by the three methods currently supported by the PDB. Of the three, 3DEM shows the greatest growth rate.

The complexity of structures archived in the PDB has increased over time, starting from the single-chain structures of myoglobin to more complex macromolecular assemblies such as the ribosome and viruses. There are now more than 600 full ribosome structures and several structures of viruses including Zika, Ebola, dengue and enteroviruses (Rossmann, 2013 ▸; Kaelber *et al.*, 2017 ▸). Most notably the RCSB PDB has set up a resource page for the 2019-nCoV (coronavirus) related structures (https://www.rcsb.org/news?year=2020&article=5e74d55d2d410731e9944f52&feature=true).

**Figure 4:**
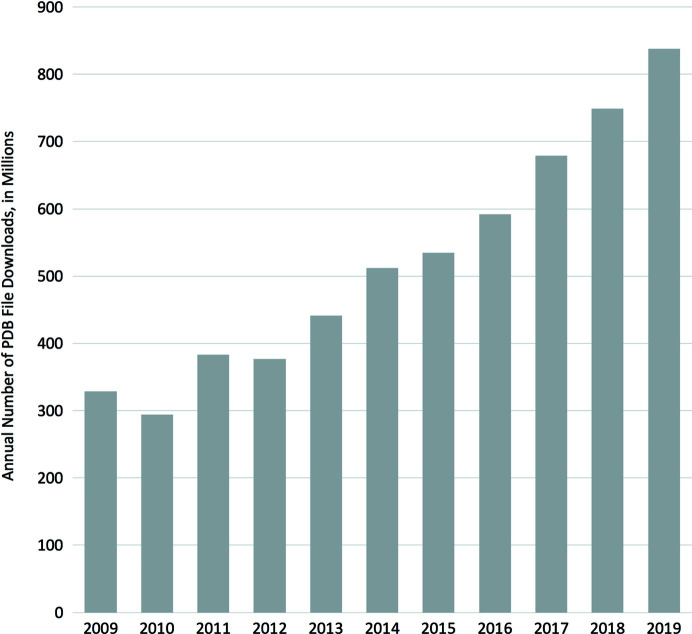
Total annual downloads of PDB archive files. Plotted values represent the sum of annual downloads from all of the wwPDB partner ftp and websites. Data Source: https://www.wwpdb.org/stats/download.

The usage of the PDB is remarkable, with 900 million downloads in 2019 (Fig. 4 ▸). These structures are used in many ways including as starting models for crystal structures being solved by molecular replacement and for fitting 3DEM maps. Modelers make particularly heavy use of the PDB. For example, the CASP project uses PDB data to develop methods for structure prediction (Kryshtafovych *et al.*, 2019 ▸). Biochemists and biophysicists use structures to help explain their findings and structures in the PDB have facilitated the discovery of several new drugs (Westbrook & Burley, 2019 ▸). The wwPDB partners maintain heavily accessed websites that offer many scientific and educational services.

## 3. Other structural biology databases   

As the field of structural biology grew, new data resources were developed to complement and supplement the data in the Protein Data Bank. These include repositories for new types of primary data and knowledgebases that integrate data from resources in other fields of biology. A summary of some of these resources is given here.

The Nucleic Acid Database (NDB) (Berman *et al.*, 1992 ▸) was originally developed as a resource for annotated nucleic acid structures. Although the PDB did accept nucleic acid structures, the focus of annotation was on proteins. In the late 1990s, an agreement was reached with the PDB for the NDB to do the primary annotation on nucleic acid structures and transfer them to the PDB. The NDB also became the proving ground for developing the mmCIF standard. The internal format for the NDB was mmCIF, which allowed the data to be easily loaded into a relational database. When the management of the PDB moved to RCSB, the NDB became a knowledgebase used by specialists in nucleic acids. It contains annotations for the nucleic acid base pairs, backbone conformations and structural motifs as well as functional descriptions of proteins bound to nucleic acids.

Recognizing that publicly available 3D density maps could accelerate discovery in structural biology and medicine, the Electron Microscopy Data Bank (EMDB) at the European Bioinformatics Institute (EBI) was launched in 2002 (Henrick *et al.*, 2003 ▸). EMDB accepts maps determined using any cryo-EM method, including single-particle reconstruction with any symmetry, helical filament reconstruction, subtomogram averaging, tomography, electron crystallography and micro electron diffraction, along with metadata describing the full experimental workflow.

**Figure 5:**
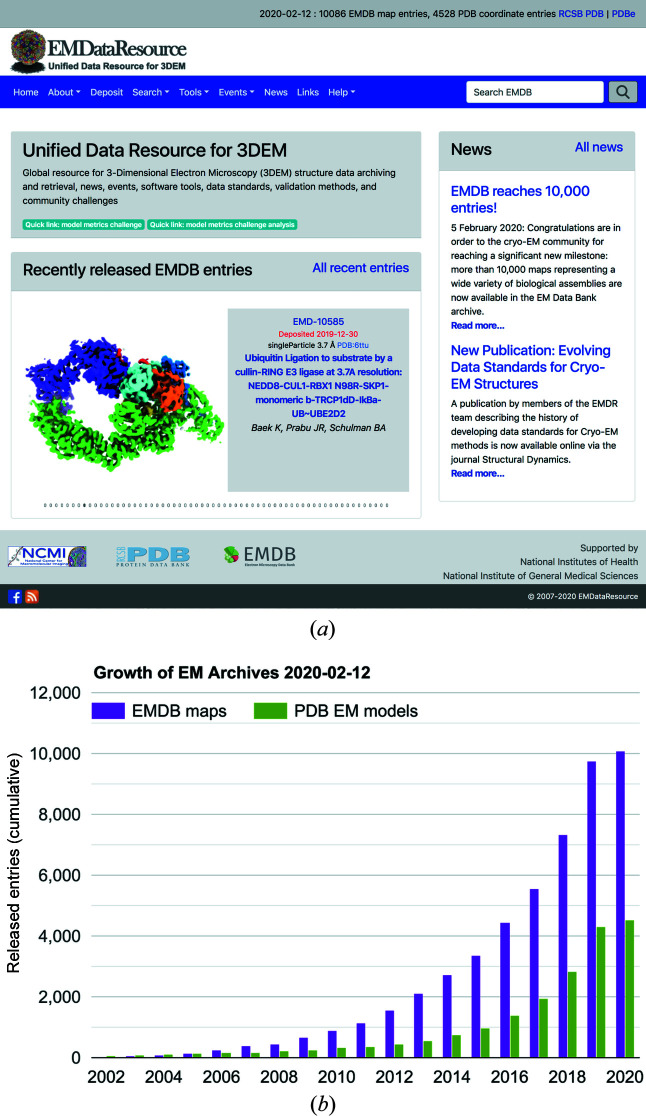
(*a*) Recent screenshot of the EMDataResource website (https://www.emdataresource.org). The website is updated weekly to highlight all newly released EMDB maps. (*b*) Cumulative number of 3DEM maps available in the EMDB and coordinate models available in the PDB by year; 2020 statistics are through February 2. Source: https://www.emdataresource.org/statistics.html.

In 2006, scientists from the EMDB, RCSB and the National Center for Macromolecular Imaging (NCMI) initiated a collaboration to ensure that data archiving and validation standards for cryo-EM maps and models are coordinated internationally (Lawson *et al.*, 2011 ▸). The project, now known as EMDataResource (EMDR; https://emdataresource.org) hosted the first 3DEM VTF (Henderson *et al.*, 2012 ▸). The EMDR project website [Fig. 5 ▸(*a*)] serves as a global resource for cryo-EM structure data and EM-related news, events, software tools, data standards, validation methods and community challenges (Lawson *et al.*, 2016 ▸; Lawson & Chiu, 2018 ▸). The site also offers growth statistics for 3DEM structures in the PDB and maps in the EMDB [Fig. 5 ▸(*b*)].

In 2012, the Electron Microscopy Public Image Archive (EMPIAR) was established at EBI (Iudin *et al.*, 2016 ▸). EMPIAR enables cryo-EM scientists to archive and share raw images and intermediate data files associated with their maps deposited into the EMDB. Making raw image data broadly available has multiple benefits, including accelerating development of reconstruction software and enriching resources for cryo-EM scientists in training. Approximately 4% of the EMDB entries deposited since 2012 have associated EMPIAR entries.

Creation of a publicly available database for experimental NMR data was first proposed in 1989 (Ulrich *et al.*, 1989 ▸). The design and implementation of the NMR database called BioMagResBank (BMRB) began in 1991 (Seavey *et al.*, 1991 ▸). BMRB is a repository for data obtained from NMR spectroscopy experiments carried out on biological systems (Ulrich *et al.*, 2008 ▸; Romero *et al.*, 2020 ▸) and employs the NMR-STAR (Ulrich *et al.*, 2019 ▸) data standards to describe NMR experiments as well as many kinds of NMR spectral data and derived data (*e.g.* assigned chemical shifts, restraints, coupling constants, relaxation parameters, *etc*.). The BMRB became a core member of the wwPDB in 2007 (Markley *et al.*, 2008 ▸), allowing for common practices to be established for depositions of biomolecular NMR data in the BMRB and the associated structural models in the PDB. Currently, about 10% of structures deposited in the PDB have been determined using NMR spectroscopy. An extension of PDBx/mmCIF, called NMR Exchange Format (NEF) (Gutmanas *et al.*, 2015 ▸) has been created to facilitate data exchange.

Small angle scattering (SAS) of X-rays and neutrons provides information regarding 3D structures and structural changes of biomacromolecules in solution. Recent advances have led to the use of SAS in conjunction with X-ray diffraction, NMR and 3DEM as a complementary method to determine the structures of macromolecules. In 2013, the wwPDB set up an SAS Validation Task Force (SASVTF) to address the requirements for archiving SAS data (Trewhella *et al.*, 2013 ▸). Following the SASVTF recommendations (Trewhella *et al.*, 2013 ▸), the Small Angle Scattering Biological Data Bank (SASBDB) (Valentini *et al.*, 2015 ▸) was established in 2015 at the European Molecular Biology Laboratory, Hamburg Outstation. The SASBDB is a curated repository for data obtained from SAS experiments. The archival standards for the SASBDB are encoded in the sasCIF data dictionary (Malfois & Svergun, 2000 ▸), an extension of the PDBx/mmCIF data representation. The sasCIF dictionary describes SAS experimental data, SAS derived models and additional metadata required for analysis and validation (Kachala *et al.*, 2016 ▸).

Structures of complex macromolecular assemblies are increasingly determined using integrative modeling (Rout & Sali, 2019 ▸), where a combination of complementary experimental and computational techniques is employed. In addition to traditional structure determination methods such as X-ray diffraction, NMR and 3DEM, experimental techniques such as SAS, atomic force microscopy (AFM), chemical cross-linking (CX), mass spectrometry (MS), hydrogen/deuterium exchange (HDX), Förster resonance energy transfer (FRET), electron paramagnetic resonance (EPR), and various proteomics and bioinformatics approaches contribute to integrative modeling. Spatial restraints derived from the different kinds of experimental and computational methods are combined to determine integrative structures of the macromolecular assembly. In 2014, the wwPDB established an Integrative/Hybrid Methods (IHM) Task Force and sponsored a workshop that engaged a community of experts to address the challenges involved in archiving integrative structures. A white paper was published (Sali *et al.*, 2015 ▸) with recommendations for archiving integrative structures.

**Figure 6:**
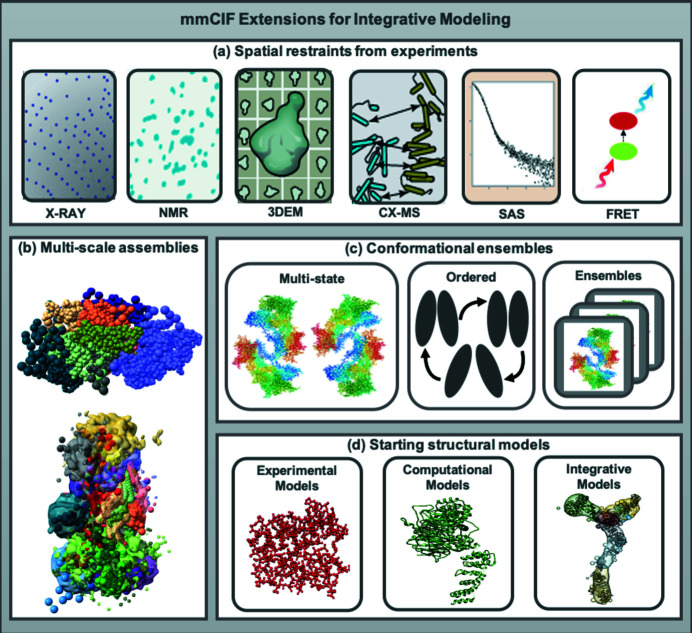
The IHM dictionary provides definitions for (*a*) spatial restraints from experimental methods such as X-ray diffraction, NMR, 3DEM, CX-MS, SAS and FRET; (*b*) multi-scale assemblies consisting of both atomic coordinates and coarse-grained representations; (*c*) ensembles representing multiple conformational states or ensembles related by time or other criteria such as events in a sequential pathway; and (*d*) starting structural models used in integrative modeling.

Based on the recommendations of the wwPDB IHM Task Force, an IHM extension of the PDBx/mmCIF dictionary (Fig. 6 ▸) has been developed to describe integrative structures and their associated spatial restraints (Vallat *et al.*, 2018 ▸, 2019 ▸). The IHM dictionary extension contains definitions for multi-scale models with atomic and coarse-grained representations, ensembles in multiple conformational states, spatial restraints derived from different kinds of experimental techniques, starting structural models used in integrative modeling and simplified definitions of the modeling workflow.

**Figure 7:**
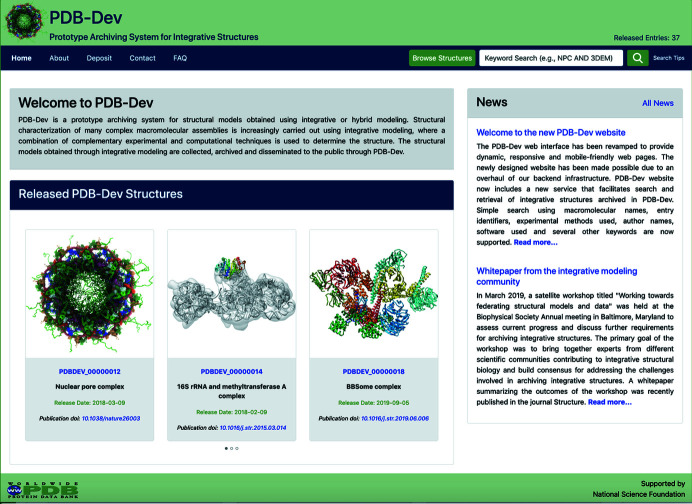
Screenshot of the current PDB-Dev website (https://pdb-dev.wwpdb.org). PDB-Dev currently consists of over 40 integrative structures including several that are on hold for publication. The structures archived in PDB-Dev vary in complexity from simple atomic structures in a single conformational state to complex coarse-grained assemblies in multiple conformational states. The data model underlying PDB-Dev supports the representation of these complex structures as well as the diverse set of spatial restraints used in building them.

A prototype archiving system PDB-Dev (Fig. 7 ▸) has been created to archive integrative structural models (https://pdb-dev.wwpdb.org) (Burley *et al.*, 2017 ▸; Vallat *et al.*, 2018 ▸, 2019 ▸). PDB-Dev was built based on the definitions in the IHM dictionary and consists of about 40 integrative structures of macromolecular complexes as of March 2020.

In 2019, a Biophysical Society (BPS) satellite workshop assessed progress and discussed further requirements for archiving integrative structures. One of the recommendations that emerged was the development of common data standards to enable efficient data exchange among the scientific repositories contributing to structural biology (Berman *et al.*, 2019 ▸). The recommendations provide the foundation for building a global federation of interoperating scientific resources that follow common data management practices and enable efficient data sharing and archiving.

Following the workshop, practitioners of several different experimental methods have engaged in further community-building activities. For instance, the HDX-MS community has published a white paper with recommendations for performing, interpreting and reporting HDX-MS experiments (Masson *et al.*, 2019 ▸), the CX-MS community is in the process of finalizing their recommendations with regards to standards and archiving of CX-MS data, the FRET community has established a platform for joint scientific efforts in the field of FRET (https://www.fret.community), the 3DEM community is working on recommendations for validating 3DEM maps and models, and the integrative modeling community is focused on building a comprehensive infrastructure for PDB-Dev and creating methods for validating integrative structures.

## 4. Perspectives   

In this review we show how the crystallographic community has played a leadership role in establishing data standards and creating an effective framework for responsible data management. The PDB has set an example for bottom-up, community-driven establishment of data management practices, paving the way for the development of standards and for the creation of several other structural biology resources. Now other biological communities that contribute to integrative structural biology are coming together to develop data standards and promote data sharing. This steady progression ensures that, in time, there will be a global network of interoperating data resources that enable scientific research. Given this trajectory, it is not overly optimistic to speculate that, in the next decade, it will be possible to tackle very large structure determination challenges such as the creation of a spatio-temporal model of an entire cell (Singla *et al.*, 2018 ▸).
